# Pharmacokinetics of recombinant human growth hormone administered by cool.click™ 2, a new needle-free device, compared with subcutaneous administration using a conventional syringe and needle

**DOI:** 10.1186/1472-6904-7-10

**Published:** 2007-10-08

**Authors:** Chris Brearley, Anthony Priestley, James Leighton-Scott, Michel Christen

**Affiliations:** 1 Clinical Research, Serono International SA, 1211 Geneva, Swizerland; 2 LCG Bioscience, Bourn Hall, Bourn, Cambridge CB3 7TR, UK; 3 Patients Care Technologies Center of Expertise, Merck Serono International SA, 1211 Geneva, Switzerland

## Abstract

**Background:**

Growth hormone (GH) is used to treat growth hormone deficiency (GHD, adult and paediatric), short bowel syndrome in patients on a specialized diet, HIV-associated wasting and, in children, growth failure due to a number of disorders including Turner's syndrome and chronic renal failure, and in children born small for gestational age. Different brands and generic forms of recombinant human growth hormone (r-hGH) are approved for varying indications in different countries. New ways of administering GH are required because the use of a needle and syringe or a device where a patient still has to insert the needle manually into the skin on a daily basis can lead to low adherence and sub-optimal treatment outcomes. The objective of this study was to assess the relative bioavailability of r-hGH (Saizen^®^, Merck Serono) administered by a new needle-free device, cool.click™ 2, and a standard needle and syringe.

**Methods:**

The study was performed with 38 healthy volunteers who underwent pituitary somatotrope cell down-regulation using somatostatin, according to a randomized, two-period, two-sequence crossover design. Following subcutaneous administration of r-hGH using cool.click™ 2 or needle and syringe, pharmacokinetic parameters were analysed by non-compartmental methods. Bioequivalence was assessed based on log-transformed AUC and C_max _values.

**Results:**

The 90% confidence intervals for test/reference mean ratio of the plasma pharmacokinetic variables C_max _and AUC_0-inf _were 103.7–118.3 and 97.1–110.0, respectively, which is within the accepted bioequivalence range of 80–125%. r-hGH administered by cool.click™ 2 is, therefore, bioequivalent to administration by needle and syringe with respect to the rate and extent of GH exposure. Treatment using cool.click™ 2 was found to be well tolerated. With cool.click™ 2 the t_max _was less (3.0 hours) than for needle and syringe delivery (4.5 hours), p = 0.002 (Friedman test), although this is unlikely to have any clinical implications.

**Conclusion:**

These results demonstrate that cool.click™ 2 delivers subcutaneous r-hGH exposure that is bioequivalent to the conventional mode of injection. The new device has the additional advantage of being needle-free, and should help to increase patient adherence and achieve good therapeutic outcomes from r-hGH treatment.

## Background

Growth hormone deficiency (GHD) affects both children and adults, and clinical manifestations vary depending on the age of onset [1]. Children present with short stature and low growth rate [2], while adults have altered body composition and metabolism with reduced physical performance [3]. At all ages, quality of life is impaired [4,5].

For many years, replacement therapy using exogenous human growth hormone (GH) has been used successfully to treat children with GHD [6], and has more recently benefited adult patients with GHD [7]. GH is now produced using recombinant DNA technology [8], and is also used to treat growth failure due to a number of other disorders including Turner's syndrome [9-11] and chronic renal failure [12], and in children born small for gestational age [13].

Conventional GH therapy for GHD was originally developed as a daily subcutaneous injection using a standard needle and syringe. However, many patients (a large proportion of who are children and adolescents) find that using needles is painful and this provokes fear of the injection procedure, resulting in potential non-adherence and sub-optimal therapy. Efforts have focused on finding alternative means of administering GH to patients. Delivery devices such as pre-filled syringes, manual injector pens, auto-injectors, injectors with hidden needles and needle-free devices have been introduced in an attempt to improve dosing accuracy and flexibility, ease-of-use, convenience, adherence and patient-friendliness [14-19]. However, the majority of injections still require manual insertion of the needle into the skin by the patient.

Needle-free devices have been introduced for GH therapy, having already been used for some time to administer insulin to patients with diabetes mellitus [20] although, in the latter case, local reactions may have limited more widespread acceptance. These devices expel the liquid preparation of the hormone through a small disposable nozzle at high pressure so that it is forced through the skin and dispersed in the subcutaneous region. This mode of administration is as effective as a conventional injection [21,22], but has the added advantage of reduced adverse psychological effects [23].

One of the available needle-free devices is the cool.click™ (cool.click^® ^in the USA), a commercially available device customized and introduced by Serono in 2000 for the purpose of injecting recombinant human growth hormone (r-hGH) with variable dosing and child-friendly ergonomic features [24]. The delivery of r-hGH by cool.click™ is bioequivalent to needle injection of r-hGH [22]. It has been shown that patients using needles and syringes to inject r-hGH had lower adherence (more patients missed over half of their prescribed dose) than those using cool.click™, resulting in significantly reduced growth rates than those who missed fewer doses [25]. Some adults and teenagers have indicated a preference for this needle-free injection device, and young children overall favoured it [22,23], reporting that cool.click™ delivery creates less discomfort than traditional needles.

In response to feedback regarding a wish for simplification of dose selection and improvement of the ergonomics of the present device, the next-generation cool.click™ 2 needle-free injection device for administration of r-hGH has now been developed by Merck Serono (an affiliate of Merck KGaA, Darmstadt, Germany). The new device is similar to the current version of cool.click™, with the additional benefit that it allows dosing in milligrams. The original cool.click™ device allowed dosing only by volume, which meant clinicians had to convert from mass (milligrams r-hGH prescribed) to volume (millilitres of solution to be injected), a procedure that could be further complicated by the fact that different volumes of solvent could be used during reconstitution of the Saizen^® ^powder for injection. In addition, the reading of the cool.click™ linear analogue dosing scale could be difficult – a vertical scale had to be aligned with a horizontal scale to set the required injection volume. In cool.click™ 2, this analogue scale has been replaced by a digital LCD dose readout. Lastly, compared with the original device, cool.click™ 2 is quieter in operation and has a modified design for ease of use and to facilitate handling by children (with smaller hands).

The main objective of this study (Study No. 25821) was to demonstrate that r-hGH administration using the cool.click™ 2 needle-free delivery device was bioequivalent to injection with a standard syringe and needle, the reference standard mode of injection.

## Methods

### Subjects

Healthy male volunteers with pituitary somatotrope cell down-regulation were screened for eligibility, for recruitment into the study. To be eligible for inclusion, subjects were required to fulfil the following criteria: age 21–50 years; have a body weight greater than 60 kg and a body mass index (BMI) in the range of 22–30 kg/m^2^; have vital signs in the normal range; and must have agreed to use barrier contraception during the study and for 3 months following completion of the post-study visit. A subject was not entered into the study if he had evidence of any surgical or medical condition that might have interfered with the pharmacokinetics of the investigational medicinal product or if he had received any investigational drug in the 12 weeks prior to dosing.

### Study design

The study was designed as a phase I, randomized, open-label, two-period, two-sequence crossover study. Treatment started within 21 days of screening. Each study period lasted 3 days, with a washout period of at least 7 days between drug administrations. The subjects were randomly assigned to one of two treatment sequences. Subjects were allocated a randomization number in sequential, chronological order immediately prior to first dose administration, in accordance with the randomization list supplied by the sponsor (Serono).

The first treatment sequence received a 0.5 mL (2.92 mg) subcutaneous dose of r-hGH (Saizen^®^, Merck Serono) administered by standard needle and syringe (period 1) followed by administration of the same dose of rhGH using the cool.click™ 2 needle-free injection device (period 2). The second treatment sequence received 0.5 mL (2.92 mg) r-hGH administered by the cool.click™ 2 device (period 1) followed by administration of the same dose of r-hGH using a standard needle and syringe (period 2).

The protocol was approved by the local research ethics committee and conducted in accordance with the Declaration of Helsinki and good clinical practice. Subjects gave written informed consent to participate in the study.

### Experimental procedures

The subjects remained in the clinical unit from 16 hours before dosing until 30 hours post-dose. To down-regulate endogenous GH sufficiently to enable accurate assessment of serum GH concentration-time profiles, somatostatin (3 mg) was given intravenously by continuous infusion for 25 hours (corresponding to a rate of approximately 1.75 μg/kg body weight/hour), commencing 1 hour prior to dosing with r-hGH to allow pituitary somatotrope cell down-regulation to be established.

Subcutaneous injections of GH were administered alternately to the left or right lower external abdominal wall with the subject in a relaxed sitting position. A different location on the external abdominal wall was used for the cool.click™ 2 needle-free injection device. The abdominal wall below the umbilicus was divided into two areas; one injection was to be administered in each area. The second injection had to be administered at least 10 cm from the first one. Each injection site was clearly circled with a permanent marker prior to dosing.

The 0.5 mL (2.92 mg) dose of r-hGH administered yielded serum hGH concentrations that remained above the limit of quantification of the hGH assay (Euro/DPC Ltd., UK; lower limit of quantification = 3.1 mIU/L) for a sufficient period to enable accurate assessment of the serum hGH concentration-time profile. Blood samples for determination of PK serum hGH concentrations were taken immediately prior to dosing and at 1, 2, 3, 4, 4.5, 5, 5.5, 6, 7, 8, 10, 12, 18 and 24 hours post-dosing in both treatment periods.

### Safety data

All clinical laboratory data outside the normal range were identified. Subject parameters including demographics and baseline characteristics, vital signs and clinical laboratory blood parameters were tabulated and assessed by descriptive statistical analyses.

The post-study examination was performed at the end of the study period 14 ± 3 days after the last dosing. Descriptive summaries were recorded for selected parameters (including demographics and baseline characteristics) using summary statistics [n, mean, standard deviation (SD), median, minimum, maximum] and frequency distributions (n, %).

Close monitoring of adverse events (AEs) was conducted throughout the study, and AEs were recorded but were not statistically evaluated. Local tolerability was assessed by inspection of the injection site at pre-scheduled time points at each period for any local reaction (redness, swelling, induration or bruising) and the severity of the reactions was evaluated.

### Data management and analysis methods

Serum concentrations of GH were analysed for each subject by non-compartmental methods using WinNonLin^® ^Professional 4.1 (Pharsight, USA).

The following pharmacokinetic parameters were computed: area under the serum concentration-time curve from time zero to the last quantifiable concentration (AUC_0-last_); area under the serum concentration-time curve extrapolated to infinity (AUC_0-inf_); peak serum concentration (C_max_); time of peak serum concentration (t_max_); and elimination half-life (t_1/2_).

The areas under the GH concentration-time curves were calculated according to the log-linear trapezoidal rule [26].

Bioequivalence was assessed according to EU Guideline CPMP/EWP/QWP/1401/98 and the FDA Code of Federal Regulations. Following logarithmic transformation, an analysis of variance (ANOVA, SAS^®^) was performed on GH metrics (C_max_, AUC_0-last_, AUC_0-inf _and t_max_) of the full analysis population. There were no imputations for missing data. The ANOVA model consisted of the logarithmically transformed C_max _parameter as the response variable with factors for sequence, subject nested in sequence, period and mode of administration (treatment). Using an average bioequivalence approach, a 90% confidence interval (CI) for the true ratio test (needle-free device) to reference (needle injection) of the means of the two treatments was produced from this model and compared with the equivalence acceptance limits 80–125%.

Based on data from previous Serono r-hGH studies, when the sample size in each sequence group is 15 (and the total sample size is 30), a crossover design has a 90% power to demonstrate equivalence within the acceptance limits of 80–125%, assuming that the expected ratio of means was 1.000, the crossover ANOVA, MSE (ln scale) was 0.250 [the SD differences, σ_d _(ln scale) were 0.354], that data were analysed in the natural log scale using t-tests for differences in means, and that each t-test was made at the 5% level. Taking into account a potential drop-out rate of approximately 20%, it was estimated that approximately 38 subjects were required to complete this study.

The pharmacokinetic analysis population consisted of all 38 subjects (100%) who were randomized into this study and who had evaluable pharmacokinetic data for both periods.

The analysis of the parameter t_max _was conducted using the non-parametric Friedman test using untransformed t_max _data.

## Results

Thirty-eight healthy male volunteers completed the study. Demographic and baseline characteristics for each subject (Table 1) were in compliance with specific inclusion and exclusion criteria. There were no major protocol deviations, no subjects dropped out and no subjects were withdrawn.

**Table 1:** Summary of baseline subject demographic data

	**Mean**	**SD**	**Range**	**n**
**Age (years)**	35.40	8.31	22.0; 49.0	38
**Height (m)**	177.80	6.15	160.0; 188.0	38
**Weight (kg)**	80.67	7.49	66.5; 97.5	38
**BMI (kg/m^2^)**	25.50	1.82	22.2; 29.8	38

BMI, body mass index; SD, standard deviation

**Figure 1:**
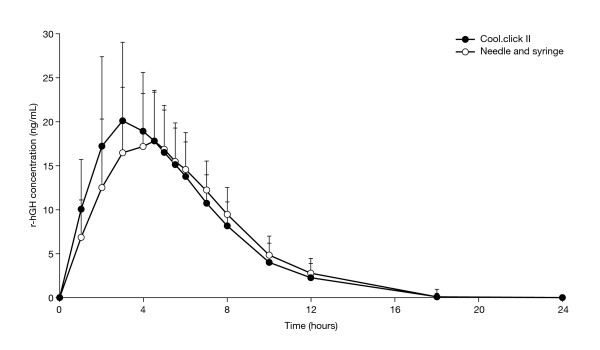
Mean hGH (± SD) serum concentration vs time profiles following subcutaneous administration of 2.92 mg rhGH/subject using either the cool.click™ 2 device or standard syringe with needle.

The ANOVA model assumptions were met satisfactorily and there was no significant sequence effect (p = 0.980). The mean ± SD serum concentration vs time profiles for GH following administration of 2.92 mg of r-hGH by either the needle-free device, cool.click™ 2, or by needle injection were generally similar throughout the 25-hour blood-monitoring period (Figure 1). Geometric mean values for AUC_0-inf_, AUC_0-last _and t_1/2 _were similar between the two administration methods (Table 2). The maximum serum GH concentrations (C_max_) of 18–20 ng/mL were observed 3–4.5 hours (t_max_) after drug administration (Table 2, Figure 1).

**Table 2:** Mean (CV%) pharmacokinetic parameters after subcutaneous administration of 2.92 mg r-hGH/subject by cool.click™ 2 or needle injection

	**cool.click™ 2 (test) mean (CV%)**	**Needle and syringe (reference) mean (CV%)**	**Ratio (test/ref)**	**90% CI for ratio**	**Falls within 80–125% range?**	**p-value**
**AUC_0-last _(hours·ng/mL)**	125.1 (30)	119.3 (27)	104.8	98.1, 112.0	Yes	
**AUC_0-inf _(hours·ng/mL)**	135.5 (27)	131.1 (24)	103.4	97.1, 110.0	Yes	
**C_max _(ng/mL)**	20.0 (43)	18.0 (37)	110.8	103.7, 118.3	Yes	
t_max _(hours)*	3.0 (2–6)	4.5 (2–6)				0.002
**t_1/2 _(hours)**	2.17 (42)	2.26 (37)				

CV, coefficient of variation; AUC_0-last_, area under the serum concentration-time profile from time zero to the last quantifiable concentration; AUC_0-inf_, area under the serum concentration-time profile from time zero to infinity; C_max_, maximum serum concentration; t_max_, time that C_max _occurs; t_1/2_, elimination half-life. Means are geometric least square means, n = 38

* Median (range)

The 90% CIs for the ratio of test (new cool.click™ 2 device) to reference (standard syringe with needle) expressed as a percentage for AUC_0-last _(98.1, 112.0), AUC_0-inf _(97.1, 110.0) and C_max _(103.7, 118.3) were all within the specified acceptance range (80–125%) for average bioequivalence (Table 2).

The median t_max _following dosing with the needle and syringe was 4.5 hours, compared with 3 hours following dosing using the cool.click™ 2 device (Table 2), and Friedman testing showed that this difference was statistically significant (p = 0.002).

No serious or life-threatening AEs were observed, and no subject was withdrawn due to AEs. The majority of AEs were of mild intensity and short duration, most frequently headache (needle and syringe, n = 11 [20%]; cool.click™ 2, n = 9 [13.2%]), and nausea of mild severity (needle and syringe, n = 10 [18.2%] and cool.click™ 2, n = 12 [17.6%]). Although there was a higher incidence of local redness in subjects after use of the cool.click™ 2 device compared with the use of needle and syringe (14 vs 3 affected subjects, respectively), this was generally mild and was not associated with any significant difference in pain, bruising, swelling, induration or itching. Redness after the use of cool.click™ 2 was most prevalent between the 5 minute and 4 hour assessments, but was experienced at each of the scheduled assessments. The incidence of redness was highest at the 5 minute and 4 hour assessments (14 subjects (36.8%) and 9 subjects (23.7%), respectively). Five subjects experienced pain after dosing with needle and syringe and 3 experienced pain after dosing with cool.click™ 2.

In this study, statistical assessment of the extent and rate of absorption of r-hGH shows that administration by a new needle-free device, cool.click™ 2, is bioequivalent to standard needle injection and demonstrates similar good tolerability.

## Discussion

The results of this study demonstrate that the concentration-time profile of hGH following subcutaneous delivery of r-hGH using the cool.click™ 2 needle-free injection device is comparable to the concentration-time profile of the same dose of r-hGH administered subcutaneously by a needle and syringe. Regulatory guidance stipulates that the 90% CIs for the ratios (test to reference) of the areas under the serum concentration vs time curves (AUC ratio) and the maximum plasma drug concentrations (C_max _ratio) must fall between 80% and 125% [27], and this study shows that both the rate and extent of exposure of r-hGH meet the accepted criteria for bioequivalence. These criteria have been used for many years, and are the same for all drugs and routes of administration.

For the purposes of establishing bioequivalence, cool.click™ 2 was compared with needle and syringe delivery, which is considered to be the standard reference for bioequivalence assessment of GH therapies. Its predecessor, cool.click™, was previously proven to be bioequivalent to this reference using similar methods [22], and this approach has also been used for another jet injection device [28].

The earlier time to maximum plasma concentration (t_max_) for the cool.click™ 2 device compared with needle and syringe delivery is unlikely to have any clinical implications on the chronic dosing regimens used for GH therapy. This is because the r-hGH therapeutic effect is not directly related to t_max _as is often the case with a single-dose therapy, for example a hypnotic used to induce sleep. A possible explanation for the difference in t_max _might be the nature of the jet injection, which tends to administer the drug deeper than by needle and with a wider spread and, therefore, the hormone is dispersed faster and absorbed into the blood faster. A similar observation has also been recorded with another jet injection device [28].

Although no statistical analysis was performed to compare AEs between the cool.click™ 2 device and needle and syringe methods of administration of r-hGH, both were well tolerated in this study in terms of both AEs and local tolerability and pain assessment. Local redness, generally mild, was the most commonly observed reaction after administration of r-hGH by needle and syringe and by cool.click™ 2. Subjects made no complaints about the device.

Many factors influence patient adherence to GH therapy, and are being addressed by needle-free technology, including the anxiety associated with a 'fear of needles', as well as occupational needle-related injuries. In addition, the functionality and appearance of the delivery device are of key importance for widespread acceptance. The additional aesthetic and ergonomic benefits incorporated into the design of cool.click™ 2 mean that it has the potential to be even more patient-friendly than currently-used devices.

## Conclusion

In conclusion, cool.click™ 2 delivers subcutaneous r-hGH exposure that is bioequivalent to exposure following injection by a needle and syringe, but has the additional advantage of being needle-free, making it particularly suitable for children and adults with an aversion to needles. By increasing patient adherence, cool.click™ 2 should help to achieve good therapeutic outcomes for GHD and other disorders that benefit from administration of exogenous GH.

## Competing interests

CB was and MC is an employee of Merck Serono. AP is and JL-S was an employee of LCG Bioscience, which was engaged by Serono to perform this study.

## Authors' contributions

All the authors were involved in the conduct of the study, all critically reviewed and revised the manuscript and all have given final approval of the submitted version.

## Pre-publication history

The pre-publication history for this paper can be accessed here:


